# Does Sex Speed Up Evolutionary Rate and Increase Biodiversity?

**DOI:** 10.1371/journal.pcbi.1002414

**Published:** 2012-03-08

**Authors:** Carlos J. Melián, David Alonso, Stefano Allesina, Richard S. Condit, Rampal S. Etienne

**Affiliations:** 1 National Center for Ecological Analysis and Synthesis, University of California, Santa Barbara, California, United States of America; 2 Center for Ecology, Evolution and Biogeochemistry, Swiss Federal Institute of Aquatic Science and Technology, Kastanienbaum, Switzerland; 3 Community and Conservation Ecology Group, University of Groningen, Groningen, The Netherlands; 4 Department of Ecology and Evolution, University of Chicago, Chicago, Illinois, United States of America; 5 Center for Tropical Forest Science, Smithsonian Tropical Research Institute, Balboa, Ancon, Republic of Panama; ETH Zurich, Switzerland

## Abstract

Most empirical and theoretical studies have shown that sex increases the rate of evolution, although evidence of sex constraining genomic and epigenetic variation and slowing down evolution also exists. Faster rates with sex have been attributed to new gene combinations, removal of deleterious mutations, and adaptation to heterogeneous environments. Slower rates with sex have been attributed to removal of major genetic rearrangements, the cost of finding a mate, vulnerability to predation, and exposure to sexually transmitted diseases. Whether sex speeds or slows evolution, the connection between reproductive mode, the evolutionary rate, and species diversity remains largely unexplored. Here we present a spatially explicit model of ecological and evolutionary dynamics based on DNA sequence change to study the connection between mutation, speciation, and the resulting biodiversity in sexual and asexual populations. We show that faster speciation can decrease the abundance of newly formed species and thus decrease long-term biodiversity. In this way, sex can reduce diversity relative to asexual populations, because it leads to a higher rate of production of new species, but with lower abundances. Our results show that reproductive mode and the mechanisms underlying it can alter the link between mutation, evolutionary rate, speciation and biodiversity and we suggest that a high rate of evolution may not be required to yield high biodiversity.

## Author Summary

The role of sex in driving genetic variation and the speed at which new species emerge has been debated for over a century. There is experimental and theoretical evidence that sex increases genetic variation and the speed at which new species emerge, although evidence that sex reduces variation and slows the formation of new species also exists. Surprisingly, given the link between sex and genetic variation, little work has been done on the impact of sex on biodiversity. In the present theoretical study we show that a faster evolutionary rate can decrease the abundance of newly formed species and thus decrease long-term biodiversity. This leads to the paradoxical result that sexual reproduction can increase genetic variation but reduce species diversity. These results suggest that reducing the rate of appearance of genetic variation and the speed at which new species emerge may increase biodiversity in the long-term. This unexpected link between reproductive mode, the speed of evolution and biodiversity suggests that a high evolutionary rate may not be required to yield a large number of species in natural ecosystems.

## Introduction

The impact of sexual reproduction on the rate of evolution could stand as one of biology's grand achievements [1]–[4]. Does sex speed genetic divergence, speciation, and thus increase the world's diversity relative to asexual reproduction? An immediate difficulty with any theory is how to define speciation in asexual organisms, where Mayr's Biological Species Concept [5] does not easily apply [6], [7]. Nevertheless, asexual organisms do diversify and are assigned species names [8]–[12], and many observations and experiments describe speciation in sexual as well as asexual organisms. Much work emphasizes ecological divergence and speciation [13]–[17], but we propose to step back and ask basic questions about the dynamics of divergence and extinction, and how it depends on sexual reproduction. Before we understand the full impact of sex on evolution and diversity in an ecologically complex world, we need to understand well the basic dynamics of mutation, gene flow, drift and extinction underlying the process of speciation.

Sex increases the rate of evolution [18]–[24], although evidence of sex constraining genomic and epigenetic variation and slowing down evolution also exists [25]–[27]. Given these contrasting impacts of sex, the effects of reproduction mode on patterns of diversification, extinction and consequent species diversity are hard to predict, even without ecological opportunity. We here pose a basic question to connect the dynamics of sexual and asexual populations with biodiversity patterns: do sexually reproducing populations have similar biodiversity patterns as asexual populations in the absence of ecological differentiation, given equal mutation and identical definitions of the genetic divergence required for speciation? How do mutation, genetic drift, ecological drift, and gene flow act in sexual, versus asexual, populations to produce diversity?

Research on diversification of species often emphasizes the process of genetic divergence, but extinction rates are also critical. Even in the absence of selection, the dynamics of diversification and diversity may thus be influenced by mutation, genetic drift, sexual recombination, colonization, as well as population size and its role in ecological drift and extinction [17]. Other than the direct impact of sexual recombination on genetic divergence, are other aspects of the dynamics of evolution the same in sexual and asexual populations? We take here a theoretical approach to the genetics of speciation [28]–[32] in the context of neutral biodiversity theory [33]. Our goal is to model the emergence of new species using explicit genetic rules on a backdrop of individuals whose births and deaths determine abundances and extinction. This genetic model of speciation extends existing neutral models of community diversity [34]–[44] so that speciation, extinction, abundance, the population size of new species, and diversity emerge from assumptions on genetic divergence, and genetic and ecological drift. Modeling speciation as a neutral process is unrealistic, but this simplification may serve as a useful null model to compare speciation rate and species diversity in asexual vs. sexual communities, minimizing the number of assumptions about population-level patterns of speciation and extinction.

To understand the effect of reproductive mode on patterns of diversification and species diversity, we need a model describing the dynamics of genes within populations within a model of populations within a community, and we need a definition of speciation that applies to sexual and asexual populations [8], [45]. Our definition of species is used in the context of a population whose genomes diverge in a spatial landscape. In the model, a community without deme structure and no ecological differentiation [46] has 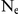 individuals, and the geographic distance between each pair of individuals 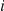 and 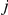 is given by 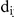; 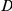 is the geographic distance matrix containing all the 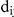 values. All individuals have identical and (essentially) infinite genomes of 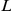 nucleotides at the outset (see “Material and Methods” and “Table 1” for a summary of the mathematical terms used in this analysis). The genetic similarity, 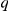, between each pair of individuals 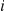 and 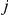 can be represented by a genetic similarity matrix, 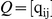. At time 0, all elements are 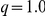, but there is a constant mutation rate 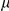 per nucleotide per birth-death cycle, so the community evolves divergence under the combined influences of recombination in sexual populations or asexual reproduction, but not both, mutation, migration, and genetic drift. We assume asexual individuals are strictly asexuals, with no horizontal gene transfer. In sexual populations, pairs mate and exchange sections of their genomes. In both models, dispersal and colonization is incorporated because offspring appear near their parents, and mating is among neighbors.

**Table 1:** Glossary of mathematical notation.

Notation	Definition
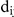	Geographical distance between individual 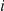 and 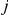
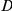	Geographic distance matrix containing all the 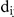 values
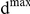	Maximum geographical distance to find a mating partner and dispersal
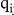	Genetic similarity between individual 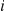 and 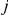
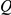	Genetic similarity matrix containing all the pairwise similarity 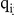 values
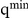	Minimum genetic similarity above which 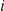 and 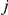 belong to the same species
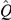	Mean genetic similarity of the matrix 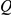 in the transient
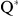	Expected mean genetic similarity of matrix 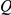 at equilibrium
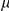	Mutation rate per nucleotide per birth-death cycle
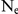	Effective population size
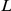	Length of the genome
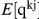	The expected genetic similarity between the new offspring 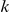 and each individual 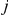 in the population
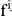	The fraction of identical sites between individual 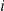 and 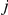
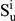	The 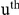 site in the genome of individual 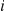
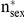	The number of direct links in a chain of inheritance before ancestor and descendant are more different than the genetic cut-off of species formation in sexual populations
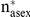	The number of direct links in a chain of inheritance before ancestor and descendant are more different than the genetic cut-off of species formation in asexual populations

**Figure 1:**
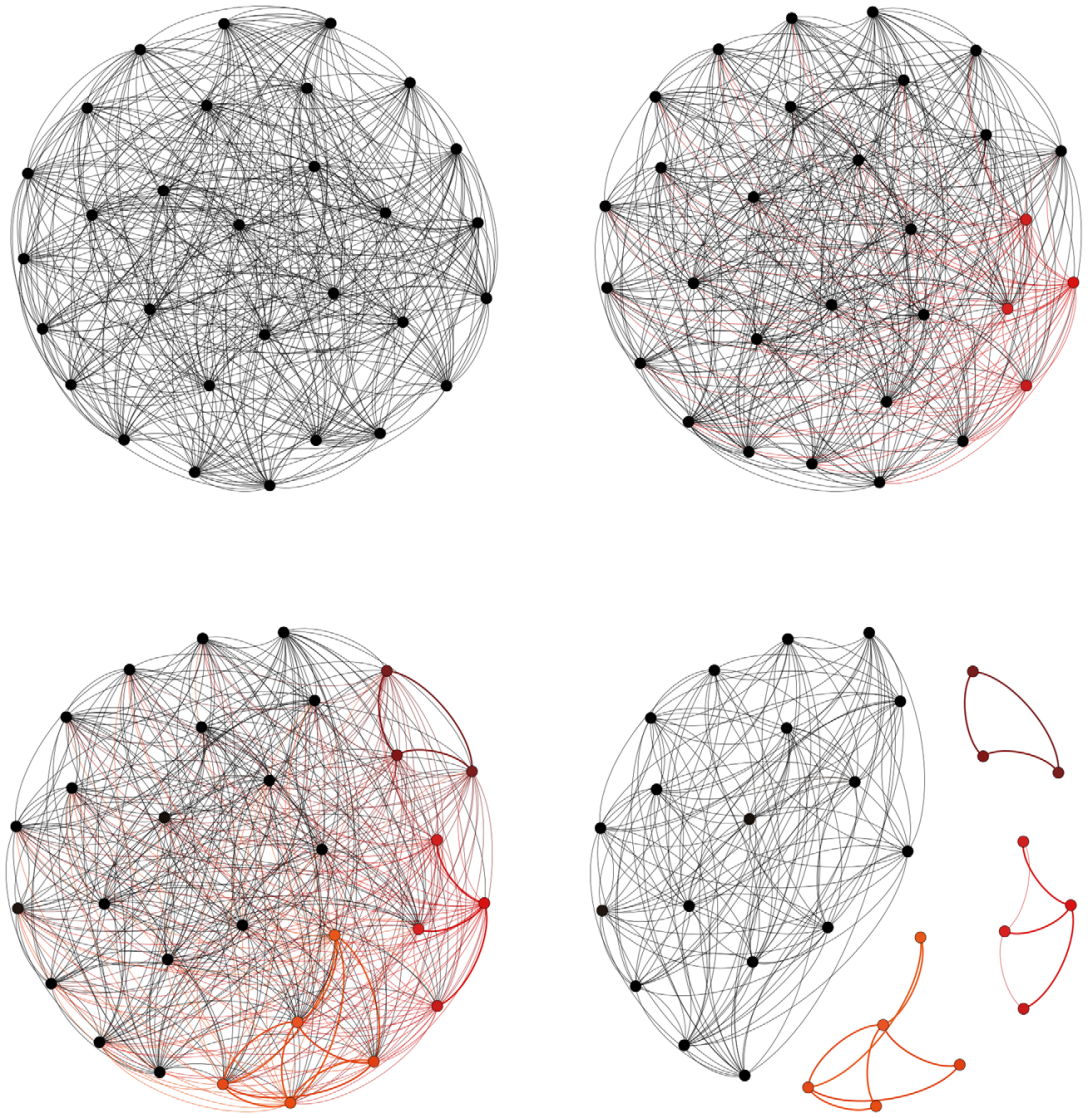
Diversification in spatial networks. **Top left**, In the initial stage all individuals, represented as black nodes, are reproductively compatible corresponding to a completely connected graph. At this stage, distance edges, represented by the geographic distance matrix, 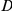, containing all the 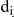 values, capture both geographical separation of each pair of individuals and viable edges. **Top right and bottom left**, Species formation start in the transients (red circles, top right and dark red, red and orange, bottom left). A species is defined as a population whose genetic similarity of each pair of individuals within the population is above a minimum genetic similarity threshold, 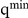. For example, the genetic similarity between each pair of individuals 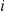 and 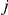, 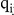, within the population in red satisfies 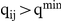. At this stage all the individuals in the network are still reproductively compatible. Formed species have different abundance (i.e, dark red (3), red (4) and orange (5). **Bottom right**, In the last stage, individuals within each species are reproductively isolated to all other individuals in the population. For example, each pair of individuals 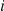 and 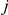 within the species in red now satisfy 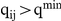 and 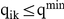 for all the individuals 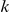 outside the population.

The crucial, final feature needed to make this a model of speciation is a minimum genetic similarity threshold, 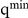 [47], [48]: two individuals 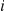 and 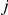 for which 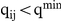 are sufficiently different to be called different species. In the sexual population, this means those two individuals cannot mate; in the asexual community, it has no impact on dynamics for the obvious reason that there is no mating. In both cases, we imagine that a biologist observing these two individuals would be inclined to describe them as different species; in the sexual case, the same biologist would detect sufficient genetic incompatibilities that offspring would be inviable [49]–[53], [53,54]. Defining critical divergence for a pair of individuals, however, is not yet a species definition, because species boundaries are a property of entire populations [31], [53], [55]. A species is defined as a connected component in a evolutionary graph: a group of individuals for which there is a path of genetic compatibility connecting every pair (Fig. 1). This means that two individuals in sexual populations can be conspecific while also being incompatible, as long as they can exchange genes indirectly through other conspecifics (a ring species [56]). Using this definition, speciation will occur whenever the expected mean genetic similarity of the matrix 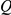 at equilibrium reaches 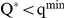 [28], [57]; intuitively, this is straightforward: with mutation too low, so 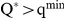, the community reaches an equilibrium similarity, 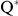, so speciation does not start (pp. 305, [57]).

In summary, the process of diversification starts with an initial phase during which genetic similarity gradually declines toward an equilibrium, 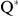. Individuals become more and more divergent from one another, particular those further away in space. Eventually, two clusters are formed with the special property that there is not a single individual in one that is compatible with any individual in the second: they form two species (Fig. 1). This is permanent, for once segregated, because given a very large genome with 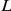 nucleotides, the universe of possible genome configurations is so large relatively to population size that it is essentially impossible for compatibility of genomes to become reestablished. The divergence process continues until each of those clusters divides further, and so on. Each speciation event leads to a loss of divergence within species, followed again by increasing divergence until another speciation event. Thus, genetic similarity within species is blocked from ever falling (much) below 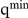 by the speciation process. But there is still more to the dynamics of diversification, due to extinction. Once we assume species formation, we must include ecological drift – random fluctuations of species abundances within a fixed metacommunity size – as an influence on dynamics, in addition to reproductive mode, mutation, migration and genetic drift. Species may start rare, or become rare due to drift, and then go extinct, and speciation should eventually be balanced by extinction, exactly as the neutral model of diversity describes [33].

We now examine this model in detail to ask whether sexual or asexual populations (and metacommunities) give rise to faster diversification or more species, considering the equilibrium, at which speciation and extinction have reached a balance, as well as the transient increase in diversity after a founding event. First, a theoretical analysis of the divergence process leads to important assertions about the speciation rate and how it relates to the mutation rate in sexual versus asexual communities. The full spatial model in which ecological drift controls the equilibrium diversity requires simulations, and we ran models with a wide variety of parameter combinations in order to answer the main questions: 1) Do species appear faster in a sexual or an asexual population? 2) Is species richness higher at equilibrium in sexual or asexual communities? 3) Are species abundances different, so does ecological drift play an important role in the extinction rate?

## Results

We first consider analytically the time course of differentiation and speciation by considering the number of steps in a chain of descendents until the threshold of genetic divergence is reached. That is the stage at which a descendent is incompatible with a founding ancestor: speciation can only happen after that. Consider asexual populations and examine one individual 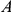 and its descendants. Denote successive individuals 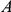 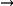 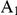 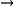 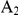,…, 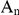, where 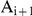 is the offspring of 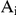. We determine the number of steps until a descendant is sufficiently different from 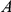 to be incompatible, so 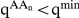. Genetic similarity between 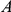 and 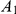 after the first offspring (following equation 18 in “Material and Methods”) is
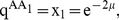
(1)where 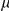 is mutation rate; after 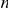 offspring, it is
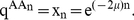
(2)The critical step, where 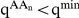, is therefore
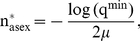
(3)A curious mutation rate is the one which produces a new cluster or species after the first offspring, so 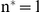, a rate so high that offspring are “hopeful monsters”: different species from their parents [58]. We call this “mutation-induced” speciation, but such a model makes little biological sense.

The model produces species with a different mechanism with much lower mutation rates: “fission-induced” speciation. Imagine a chain of descendants 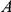 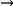 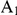 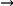 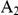,…, 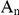 in which every individual is alive. Ignoring the “hopeful monsters” of mutation-induced speciation, the entire chain belongs to the same cluster or species, even if 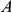 and 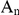 are distinct enough to be incompatible. After enough time, however, intermediate steps in the chain die, and eventually a subchain 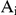 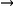 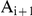 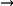 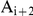,…, 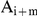 is entirely dead. Once a subchain of 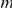 consecutive steps, with 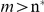, dies, the survivors in the chain become two separate species. Obviously, at some point there is a single critical individual whose death breaks the single cluster into two clusters – the last of the 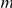 individuals in the subchain to die.

With fissioning of genetic clusters, new species need not be singletons. Indeed, there is no upper limit on the abundance of a new species (the parent population size is the upper limit). Incipient population size should depend on 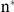, and thus the minimum genetic similarity value, 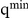, that defines a species, and mutation rate (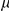): the higher 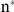, the more time it will take before we have a new cluster formation. With higher 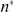, we thus anticipate lower speciation rate, but lineages may have higher incipient abundances and thus be less prone to extinction.

In an earlier work, we examined the dynamics of the number, 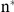, in sexual populations in the absence of a limited geographical distance for mating (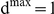) [32]. The critical number of steps where 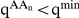 in a panmitic population with sexual reproduction is:
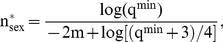
(4)The extra 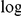 term in the denominator compared to equation 3 reflects genetic difference between mated pairs, and thus genetic dissimilarity between offspring and parents beyond mutation. Equations 3 and 4 suggest that there should be a monotonic relationship between 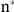 and the speciation rate. New species will form at the rate at which chains of length 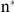 die. Comparing equations 3 and 4, we observe that 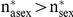 in all cases, so 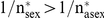. With sex, it takes fewer steps, 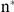, before a descendent passes the critical genetic similarity, 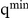, relative to its ancestor and this should lead to a lower speciation rate in an asexual metacommunity at a given mutation rate.

**Figure 2:**
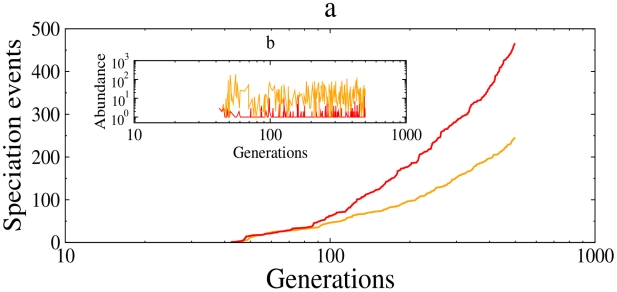
Speciation and incipient species abundance. **a**, Cumulative number of speciation events as a function of the number of generations for the sexual (red, also used for b) and asexual populations (orange also used for b). **b**, Simulated abundance of the new species after each speciation event. The plot represents the output from one replicate during the first 500 generations with mutation rate, 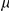 = 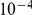, the minimum genetic similarity value, 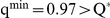, and a maximum geographic distance for mating and dispersal, 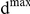 = 1.

**Figure 3:**
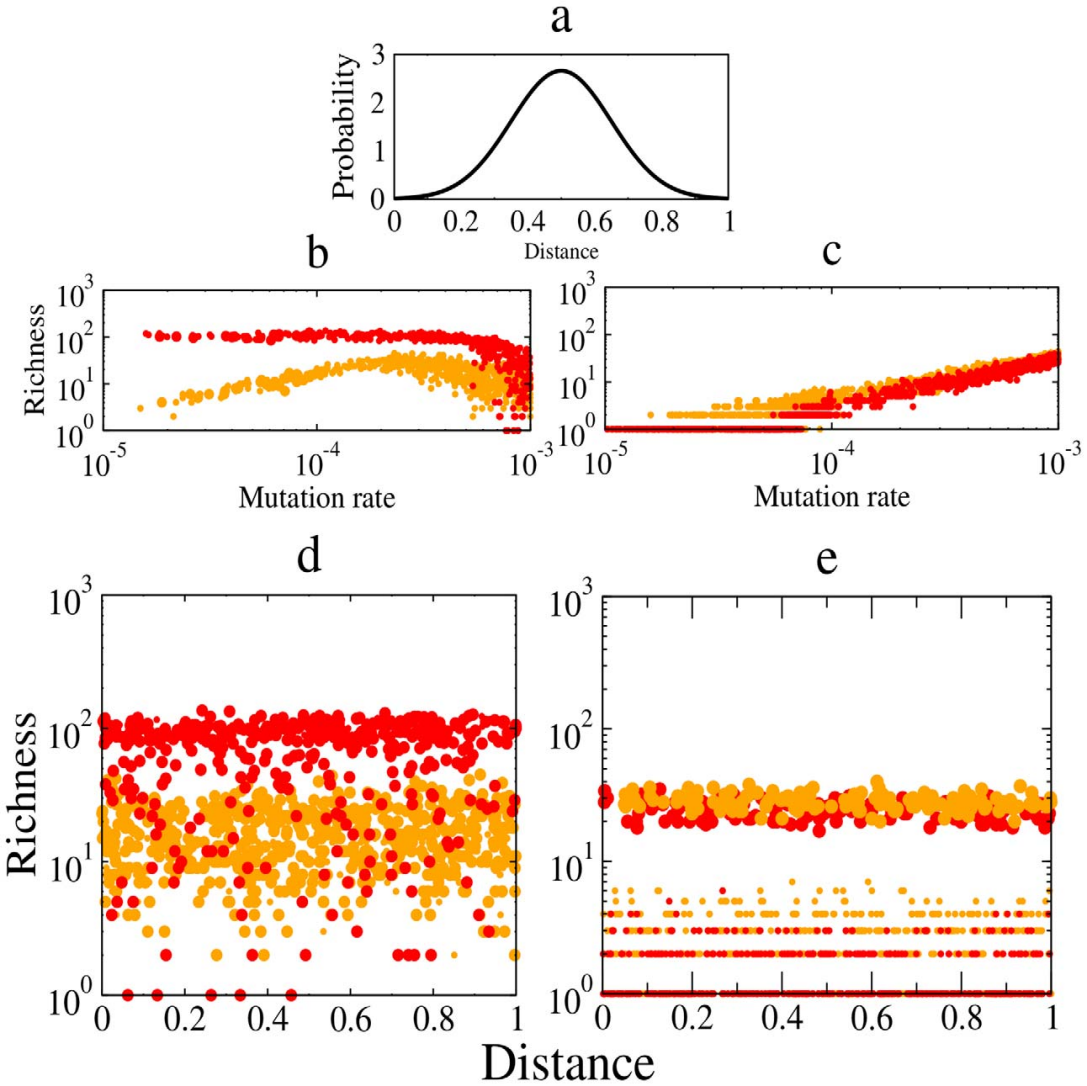
Speciation, extinction and biodiversity in spatial networks. **a**, Geographic distance between individual 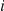 and 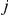, 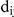, is sampled from a normal distribution, 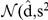). In this plot the mean, 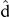 = 0.5, and the standard deviation, 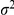 = 0.35. **b–c**, Species richness as a function of mutation rate, 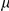, with maximum geographic distance values to find a mating partner and dispersal in the range, 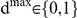 for asexual (orange) and sexual (red) populations in the transient (**b**) and last (**c**) stage. **d–e**, Species richness in the transient (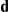) and last (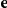) stage as a function of the maximum geographic distance to find a mating partner and dispersal, 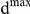 (“Distance”). The size of the circles represent the species richness at a given mutation rate in the range, 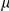 (big circles) 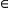 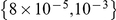 and 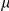 (small circles) 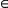 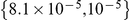. In this plot each replicate in the transients (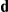) satisfies, 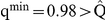 = 0.97. Sexual populations have more species in the transients for a broad range of parameter values. Richness collapses for high mutation rate values (see 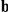), thus the high dispersion of species richness values in 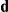. Asexual populations maintain higher biodiversity levels than the sexual populations, especially for low mutation rate values (see **c** and **e**). Each replicate in the last stage (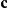 and 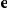) satisfies 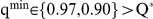.

Simulations confirm this assertion. Soon after founding, the metacommunity with sexual reproduction produces species more rapidly at a given mutation rate, 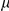, than the asexual case (Fig. 2a), but lineages have lower abundances and thus are more prone to extinction (Fig. 2b). This pattern has strong consequences for species richness (Fig. 3). In the transient, at very high mutation rate, the number of species collapsed in both models (Fig. 3b). At equilibrium, quite surprisingly, the opposite held, and the asexual model had higher number of species for low mutation rate values (Fig. 3c). The sexual model was much less efficient at maintaining species despite the higher rate of species formation. At 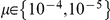, for instance, there are 1–3 species in simulations with sex, compared to 2–10 species without sex (Fig. 3c). These patterns remain the same after we compare the transient (Fig. 3d) and the steady-state (Fig. 3e) regardless of the maximum geographic distance for mating and dispersal, 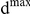.

**Figure 4:**
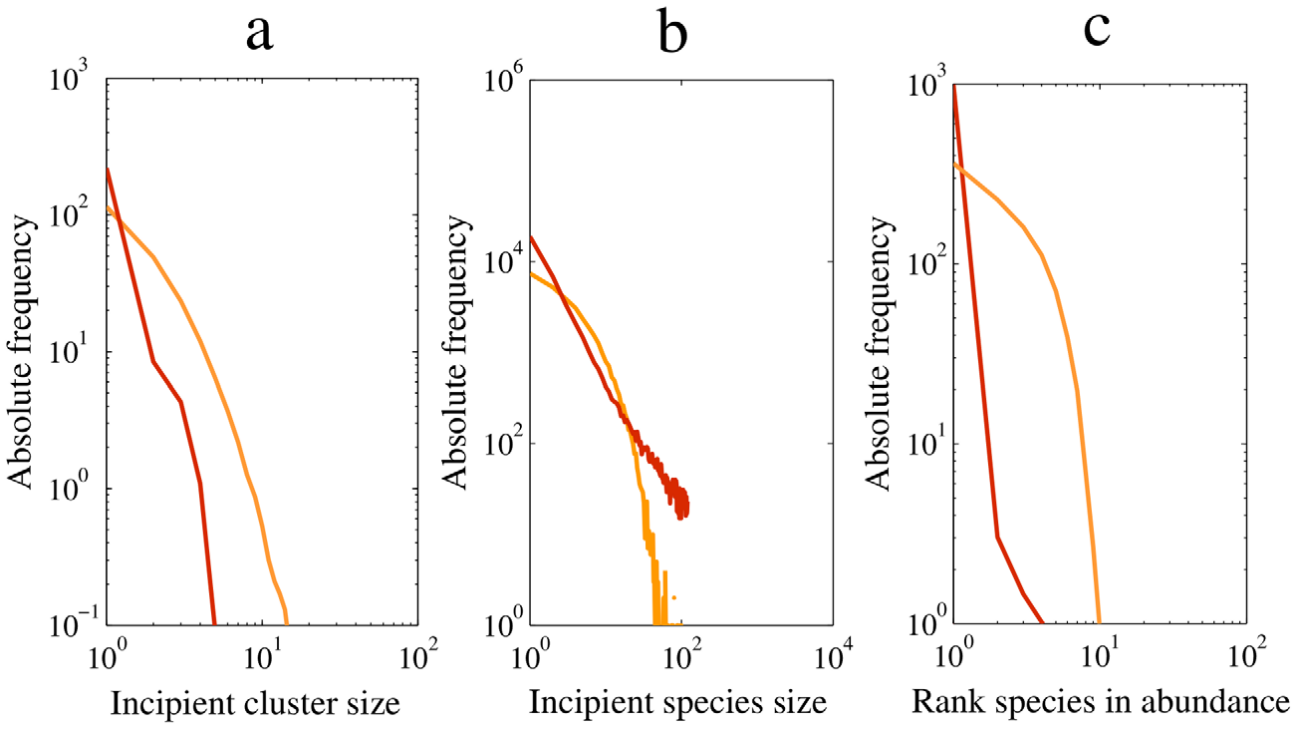
Incipient species abundance and biodiversity. **a**, Mean incipient species size distribution after 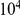 replicates in the first stage for sexual (red) and asexual (orange) populations at a given mutation, 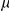 = 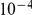. Each replicate satisfies, 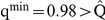 = 0.97, and a maximum geographic distance for mating and dispersal, 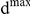 = 0.5. **b**, Mean incipient species size distribution after 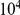 replicates in the last stage for sexual (red) and asexual (orange) populations at a given mutation, 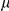 = 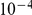. Each replicate satisfies 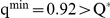 and a maximum geographic distance for mating, 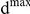 = 0.5, and **c**, Mean species abundance distribution for sexual (red), and asexual (orange) populations at a given mutation rate, 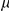 = 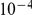, minimum genetic similarity, 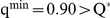, and a maximum geographic distance for mating and dispersal, 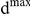 = 1.

This failure to maintain species richness in the sexual model could only have been due to extinction: at a given diversity, the sexual communities must have lost species at a higher rate than asexual communities. This would happen if incipient species abundances were more skewed in the sexual model. Fig. 4 shows that this is indeed the case. In sexual communities, there were more incipient species with low abundance in the transient (red line, Fig. 4a) and at equilibrium (red line, Fig. 4b), very few highly abundant species, and many rare species (Fig. 4c) relative to asexual communities. This pattern remains qualitatively the same in all pairwise comparisons between sexual and asexual metacommunities with 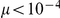 and 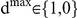 (Kolmorgorov-Smirnov test, 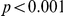).

## Discussion

In the present paper, we have explored a landscape population genetics model to understand the effect of reproductive mode on speciation and extinction rate and the connection between the abundance of new species and species richness. The approach uses processes of individual organisms with large genomes – birth, death, gene flow, mutation and genetic-ecological drift – to study macroecological patterns of biodiversity [28]–[32]. It allows a comparison of diversification rate and community diversity in sexual vs. asexual communities without recourse to any assumption about population-level patterns of speciation and extinction. By modeling speciation explicitly, genetic assumptions about the formation of species become necessary: in the present study, the constant mutation rate and threshold of genetic similarity defining the species boundary.

These assumptions allow us to derive quantitative relationships between mutation rate, abundances, probability of extinction of new species, and species richness. For example, the number of species in a metacommunity increases monotonically with mutation rate in both sexual and asexual populations. But mutation alone cannot cause speciation, because the genetic similarity defining species is also essential, entering in the equations that drive the rate of species formation (i.e., 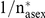 and 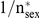). The quantitative nature of the relationship between mutation rate, genetic similarity, and species formation is understood with 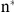: incipient population size and species richness are both functions of 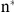, and higher 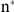 means more time between speciation events but also higher incipient abundances and lower extinction rate.

Surprisingly, sexual populations, with low 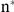 value and thus a high speciation rate, had greatly reduced species richness at equilibrium, relative to the asexual populations with otherwise similar processes. This highlights the importance of deriving the processes connecting the rate of evolution and incipient abundance – the number of individuals in newly formed clusters or species – because they both impact speciation [35], [59] and the number of species that can coexist in metacommunities. Incipient species abundance was highly variable in both sexual and asexual populations, but especially so with sex. In the latter, newly formed species were often singletons and thus rapidly went to extinction.

Most speciation events in nature are believed to have been driven by divergent selection and drift is thought to play a very small role [15], [53]. But genetic and ecological drift can be strong contributors during speciation, especially in the early stages [17]. We have shown that a higher evolutionary rate in sexual populations does not guarantee more coexisting species, especially in the long term, because higher evolutionary rate may imply a lower abundance of new species and thus a higher extinction probability. Thus, even if drift plays a small role in driving differentiation and speciation, it can strongly influence the extinction dynamics driven by the low abundance of the incipient species in natural populations.

How robust are these results after the addition of selection and ecological differentiation? Sexual organisms might have faster rates of adapting to different ecological conditions [21]–[24], [60], because multiple beneficial mutations can spread simultaneously in the population [20]. This can trigger higher abundance and lower extinction probability in sexual populations because speciation is not being driven by mutation but rather by adaptations to ecological conditions. Sex can also constrain the rate of adaptation to new conditions [25], [26]. For example, it removes major changes such as chromosomal rearrangements [27], and in the process of finding a mate, it may increase the risk to predation or higher exposition to sexually transmitted diseases [61], [62]. These processes may slow down the rates of evolution and speciation which, according to our results, may not necessarily decrease the number of species in the long term. Further research that connects genetic and ecological drift with selection in constant and fluctuating environments may shed light on the link between reproductive mode, the rate of speciation, the abundance of new species, extinction probability, and long term species richness [17], [63].

A theory that covers the link between net diversification rates and biodiversity patterns in both sexual and asexual taxa is still lacking [17], [21], [64], [65], and our approach joining population genetics models with divergence criteria and macroecological patterns of biodiversity may be a way forward. One important advance would be to develop analytical relationships among the key parameters, particular mutation rate and the strength of selection in the context of several topologies of spatial networks, and subsequently spatial heterogeneity [29], [42], [57],[66],[67]. On the genetic side, more precise consideration of the mechanisms driving genome evolution, specifically in the context of rates of self-compatibility and outcrossing, might lead to different predictions about speciation and diversity [10], [68]–[72]. We believe that our results may help to connect reproductive modes with the speciation rate in eco-evolutionary graphs, and the effect of the incipient species abundance on net diversification rate and extant diversity.

## Materials and Methods

### The models

Models of DNA evolution based on simple base pair substitution have a long history (i.e., the infinite sites model, [73], [74]), and several variants have been proposed [75]. More realistic extensions of those models include deletion, insertion, duplication and rearrangements of segments bases [70]. Recent models also take into account, as in the neutral theory of biodiversity [33], instantaneous speciation but with explicitly evolving genomes (i.e., an identical copy of one root genome is made, each of the two genomes gets a new successor species name, and they each evolve independently thereafter, see [70]).

In the models explored, the reproductive mode describes a population with evolving genomes. During asexual reproduction a mother is randomly selected while in the sexual populations, in addition to this randomly selected mother, potential mates are identified from among those within the specified geographic distance, 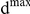. In case there are no potential mates the mother reproduces without a mate. This situation is especially relevant for the extreme case, 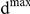 = 0. In the sexual and asexual models the offspring is then dispersed within the geographic distance, 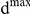, and occupies the site of a randomly killed individual within the area 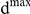. At the beginning of the simulation, all individuals are reproductively compatible, corresponding to a completely connected graph (Fig. 1). Genetic similarity among individuals in the sexual and asexual model can be represented by an evolutionary spatial graph in which nodes are individuals, distance edges capture the geographical separation of each pair of individuals and viable edges that connect individuals within the same species.

We here describe formally the derivation of equation 1 in the main text for the asexual model. The dynamics of sexual populations in the absence of dispersal limitation (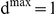) has been considered elsewhere and will not be derived here [32]. Individuals are haploid. The genome of each individual is represented by a sequence of 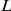 sites, each nucleotide residing in one of two states, 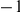 or 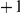. Each individual 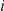 in a population of size 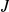 is represented as a vector: 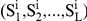, where 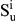 is the 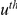 site in the genome of individual 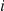. The genetic similarity between individual 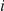 and individual 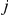 can be defined as:
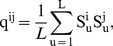
(5)with 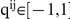. The genetic similarity in equation (5) can be written in terms of the fraction of identical sites (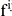)
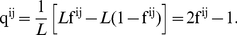
(6)and 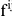 is:
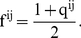
(7)


Each nucleotide in the offspring is inherited at random, thus ignoring linkage between neighboring nucleotides, but with a small probability of error determined by the mutation rate. Assuming that the individual 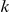 inherited the nucleotide at site 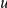 from its parent 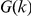 we need the probability that individual 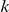 will have exactly the same nucleotide (i.e., 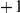 or 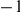) as 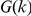. We assume that the probability of undergoing 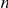 mutations in site 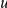 is Poisson distributed:
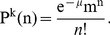
(8)Each mutation switches the nucleotide (i.e., 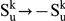). Then the probability of observing an even number of mutations, so that the nucleotide at site 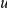 does not change the nucleotide is
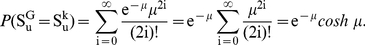
(9)The probability of an odd number of mutations, changing the nucleotide, is
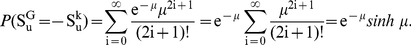
(10)


Note that we can have 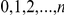 mutations in site 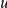 of the new offspring 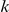, but because the mutation rate, 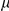, is small, most of the probability density is concentrated in the 0 and 1 point mutation cases. The probabilities can be found by solving the system:
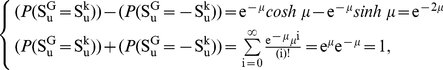
(11)thus,
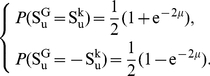
(12)This derivation is similar to those of Peliti, Serva, Higgs and Derrida [28], [76], [77], but we consider here nucleotides instead of alleles.

In the asexual model, each individual 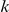 is generated by one parent, 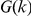. The expected fraction of nucleotides in 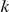 shared with each individual 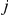 in the population (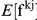) is, using equation 12,
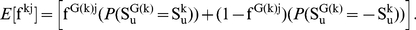
(13)Substituting 12 in 13 we have
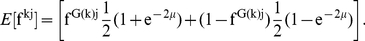
(14)Substituting 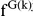 = 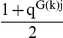 from equation 7 then gives
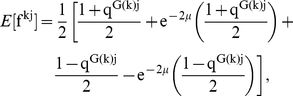
(15)and after simplification we obtain
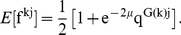
(16)Substituting equation 16 into 6 leads to
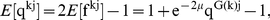
(17)and from 17 we get
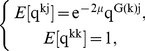
(18)and equation 3 is derived from this expectation. We used this equation to simulate the mean genetic similarity in the transients, 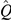 and we also used it to calculate the mean genetic similarity of the matrix, 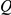, at steady-state for asexual populations, 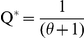, where 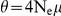 for small 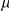 and 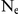 is the effective number of individuals in the population [28].

### Simulations

Our simulation is a stochastic, individual-based, zero-sum birth-death model of a sexual and asexual population with overlapping generations. For the simulations reported in the paper, we considered 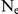 haploid individuals where only one individual can exist in each site. Simulations were carried out with an initial population, 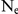 = 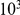 individuals, and this initial population size remained constant throughout the simulations. Results for Figs. 3 and 4 were obtained after 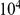 replicates and 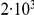 generations of a single model run, where a generation is an update of 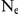 time steps. Geographic distance between each pair of individuals 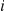 and 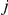, 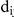, was sampled from a normal distribution, 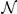 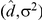 and negative values were discarded. Results were qualitatively the same after varying 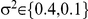. In the transients each replicate stops after the mean of the genetic similarity matrix, 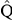, reached the values, 
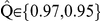
 with all the replicates satisfying 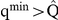 (Figs. 3b, 3d and 4a). In the last stage parameter values were chosen to satisfy the mathematical condition required for speciation, 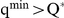 [28] (Figs. 3c, 3e and 4b–c). Steady-state was verified by checking the constancy of the mean genetic similarity value during the last 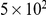 generations within each replicate regardless the initial value of 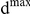. We explored a broad range of parameter combinations with mutation rate, 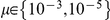, the maximum geographic distance for mating and dispersal, 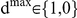, and two cut-off values to count species richness in the transient and equilibrium dynamics: the minimum genetic similarity value to define a species in the transients, 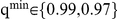, and the minimum genetic similarity value to define a species at equilibrium 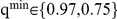, respectively.
